# Rabbit CD200R binds host CD200 but not CD200-like proteins from poxviruses

**DOI:** 10.1016/j.virol.2015.10.026

**Published:** 2016-01-15

**Authors:** Munir Akkaya, Lai-Shan Kwong, Erdem Akkaya, Deborah Hatherley, A. Neil Barclay

**Affiliations:** Sir William Dunn School of Pathology, University of Oxford, South Parks Road, Oxford OX1 3RE, UK

**Keywords:** CD200, CD200R, Myxoma virus, M141, Poxviruses

## Abstract

CD200 is a widely distributed membrane protein that gives inhibitory signals through its receptor (CD200R) on myeloid cells. CD200 has been acquired by herpesviruses where it has been shown to interact with host CD200R and downmodulate the immune system. It has been hypothesized that poxviruses have acquired CD200; but the potential orthologues show less similarity to their hosts. Myxoma virus M141 protein is a potential CD200 orthologue with a potent immune modulatory function in rabbits. Here, we characterized the rabbit CD200, CD200R and tested the CD200-like sequences for binding CD200R. No binding could be detected using soluble recombinant proteins, full length protein expressed on cells or myxoma virus infected cells. Finally, using knockdown models, we showed that the inhibitory effect of M141 on RAW 264.7 cells upon myxoma virus infection is not due to CD200R. We conclude that the rabbit poxvirus CD200-like proteins cause immunomodulation without utilizing CD200R.

## Highlights

• The coding sequences of rabbit CD200 and rabbit CD200R have been identified.
• Rabbit CD200-CD200R binding resembles CD200-CD200R interactions in other species.
• Poxvirus CD200-like molecules (M141, SFV 141) do not interact with rabbit CD200R.
• M141 can downregulate immune response without utilizing host CD200R.

## Introduction

**Fig. 1:**
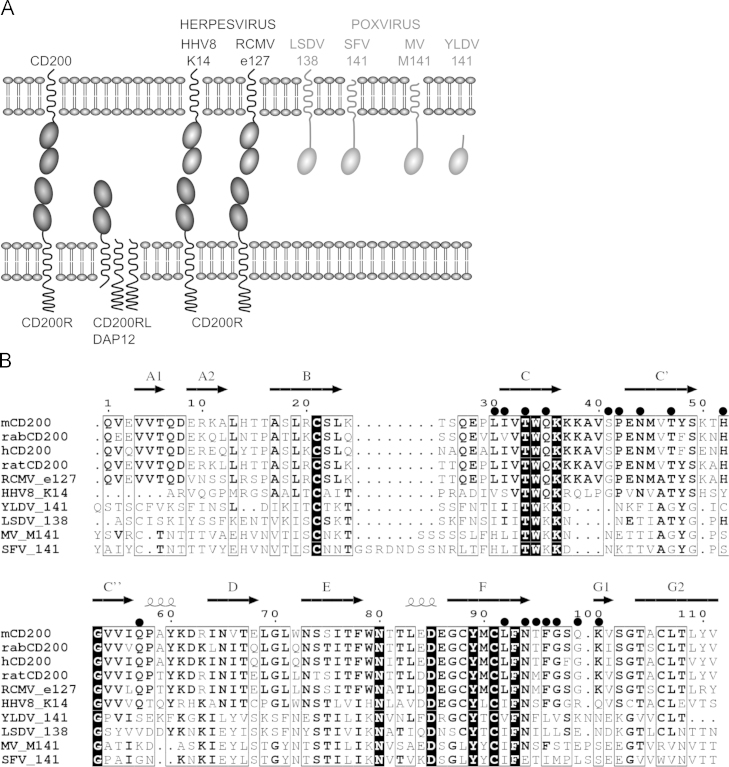
(A) Cartoon illustrating the known interactions and organization of CD200 and CD200-like proteins identified in herpes- and poxviruses. Immunoglobulin (Ig) domains are shown as ovals. CD200RL represents the activating CD200R-like proteins that associate with DAP12. Selected CD200-like proteins shown from herpesviruses are human herpes virus 8 (HHV8) and rat cytomegalovirus (RCMV). Poxvirus CD200-like proteins shown are from lumpy skin disease virus (LSDV), rabbit fibroma virus (SFV), myxoma virus (MV) and yaba-like disease virus (YLDV). (B) Amino acid sequence alignment showing domain 1 of CD200 and CD200-like proteins from selected mammals and herpes- and poxviruses. Mammalian CD200 proteins shown are mouse CD200 (mCD200, NP_034948), rabbit CD200 (rabCD200, BK009358), human CD200 (hCD200, P41217) and rat CD200 (CAA25925). CD200-like proteins from RCMV and HHV8 are e127 (AAO45420) and K14 (AAK53415) respectively. Poxvirus CD200-like proteins are YLDV 141 (NP_073526), LSDV 138 (NP_150572), MV M141 (NP_051855) and SFV 141 (NP_052027). The secondary structure of mCD200 is shown above the alignment with arrows and squiggles indicating beta sheets and alpha helices (PDB code 4BFI, chain B). Residues at the mCD200/CD200R interaction interface are denoted by black circles (Hatherley et al., 2013). Accession numbers are given in parentheses. Alignment generated using ESPript 3.0 server (Robert and Gouet, 2014).

CD200 is a broadly distributed membrane protein that can downregulate myeloid cell activity by interacting with an inhibitory receptor termed CD200R (Hoek et al., 2000). This receptor is only expressed on leukocytes with high levels on basophils and macrophages (Wright et al., 2003, Shiratori et al., 2005, Akkaya et al., 2013). A variety of herpesviruses have acquired the CD200 gene (illustrated diagrammatically in Fig. 1A) and benefit from it as a defense mechanism that downregulates the host׳s immune response (Akkaya and Barclay, 2013). The CD200 orthologues from several herpesviruses have been shown to interact with the CD200R. These include the K14 protein from human herpesvirus 8 (HHV8 or Kaposi׳s Sarcoma-associated herpesvirus (KSHV)) (Shiratori et al., 2005, Foster-Cuevas et al., 2004), the Rhesus macaque rhadinovirus (RRV) R17 protein (Langlais et al., 2006), U85 from HHV6, and HHV7 (Shiratori et al., 2005) and the e127 protein from rat cytomegalovirus (RCMV) (Foster-Cuevas et al., 2011). The HHV8 K14 protein (Foster-Cuevas et al., 2004) and the RCMV e127 protein (Foster-Cuevas et al., 2011), mimic the host protein as they have indistinguishable affinities to CD200R. Expression of the rhesus rhadinovirus (Estep et al., 2014) and HHV8 viral orthologues have been shown to downregulate myeloid activity (Shiratori et al., 2005, Foster-Cuevas et al., 2004) and HHV8 K14 can also inhibit T cell responses (Misstear et al., 2012).

CD200-like sequences are also found in many poxviruses. However the sequence similarity is limited and extends over a single domain unlike host CD200 and the herpesvirus orthologues of CD200 that contain two Ig-like domains in their extracellular regions (Fig. 1 and Table 1). The myxoma virus CD200 orthologue M141 can modulate the immune system but the hypothetical interaction with CD200R has not yet been properly tested (Cameron et al., 2005, Zhang et al., 2009). We now test whether the M141 protein can bind rabbit (*Oryctolagus cuniculus)* CD200R. *O. cuniculus* (European rabbit) is a host species close to the natural hosts of the myxoma and rabbit fibroma viruses, namely *Sylvilagus brasiliensis* (tapeti; jungle rabbit) and *Sylvilagus floridanus* (eastern cottontail) (Kerr et al., 2013, Lemos de Matos et al., 2014). While myxoma virus causes a mild cutaneous infection in *S. brasiliensis,* the infection of *O. cuniculus* results in a lethal systemic disease and M141 molecule of the virus has been shown as an important virulence factor for the development of the lethal outcome (Cameron et al., 2005). Therefore, firstly, we identified and characterized the rabbit (*O. cuniculus*) CD200 and CD200R and their interaction and secondly showed that the poxvirus CD200-like sequences, unlike the herpes CD200-like sequences, do not bind CD200R. Furthermore, through CD200R knockdown models, we have shown that the immune-modulatory action of the M141 molecule, shown previously in Zhang et al. (2009) is not related to the CD200R expression. The possible function of the viral protein and its evolution are discussed.

## Results

### Identification and analysis of rabbit CD200 and CD200R sequences

In order to be able to test whether the rabbit poxvirus CD200-like protein interacted with host CD200R, the rabbit CD200R and CD200 were identified from the genomic sequences for *O. cuniculus* by searching the Ensembl genome browser. The sequences of rabbit CD200R and CD200 showed similarity to the equivalent proteins in other mammals (e.g. 59% amino acid sequence identity with the human CD200R and 84% for human CD200).

**Fig. 2:**
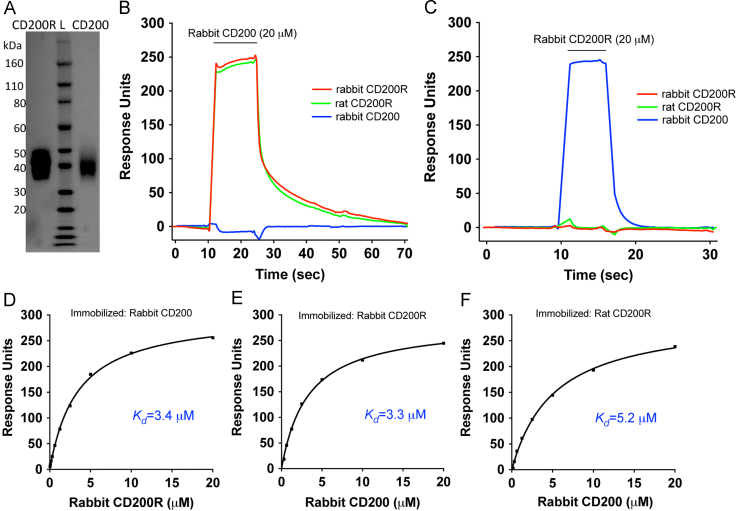
Affinity of the interaction of recombinant rabbit CD200 (Rab CD200) with rabbit CD200R (Rab CD200R). (A) Coomassie blue stained SDS PAGE showing purified proteins containing the extracellular region of rabbit CD200 and rabbit CD200R (L stands for protein ladder). (B–C) SPR analysis showing binding of soluble rabbit CD200 (B) and soluble rabbit CD200R (C) binding to rabbit CD200R rCD4d3+4, rat CD200R rCD4d3+4, rabbit CD200 rCD4d3+4. (D–F) a range of concentrations of soluble rabbit CD200R was passed over rabbit CD200 (D) and rabbit CD200 passed over rabbit CD200R (E) and rat CD200R (F) and the equilibrium coefficients for each interaction are indicated. The results are typical of three experiments.

Recombinant soluble proteins containing the extracellular domains for both receptor and ligand attached to a C-terminal His tag were expressed in CHO cells, purified by Ni affinity chromatography and gel filtration (Fig. 2A). In addition, the extracellular regions were expressed as chimeric proteins with ratCD4 d3+4 (rCD4d3+4) which is an effective and widely used antigenic tag, together with a sequence to enable site specific biotinylation (Brown et al., 1998). Recombinant purified rabbit CD200 and CD200R proteins were sequentially passed over four flow cells to which chimeric rabbit CD200, rat CD200R, rabbit CD200R and rCD4 d3+4 had been bound to streptavidin coated BIAcore CM5 chips. Clear bindings of rabbit CD200 to rabbit CD200R (and the reverse) and to rat CD200R were observed (Fig. 2B and C). The traces showed rapid binding and fast dissociation, typical of low affinity interactions such as CD200 and CD200R (Wright et al., 2003). The affinity of the rabbit CD200R/CD200 interaction was determined by passing a range of concentrations of the analytes sequentially over each protein and was found to be similar irrespective of which of the two proteins were immobilised (*K*_*d*_= 3.3–3.4 µM) (Fig. 2D and E). These affinities were comparable to those found in other species namely *K*_*d*_:1.5 µM; 0.5 µM and 4.0 µM for rat, human and mouse interactions respectively (Wright et al., 2003, Foster-Cuevas et al., 2011, Hatherley et al., 2005). The rabbit CD200 cross reacted with the rat CD200R (Fig. 2F) and the affinity (*K*_*d*_:5.1 µM) was comparable to other cross species interactions between CD200 and CD200R (Foster-Cuevas et al., 2004). These interactions have fast dissociation rates (half life of around a second) with a fast on-rate so equilibrium binding is reached rapidly (see analyses (Wright et al., 2000)).

### Myxoma M141 CD200-like protein does not bind rabbit CD200R

**Fig. 3:**
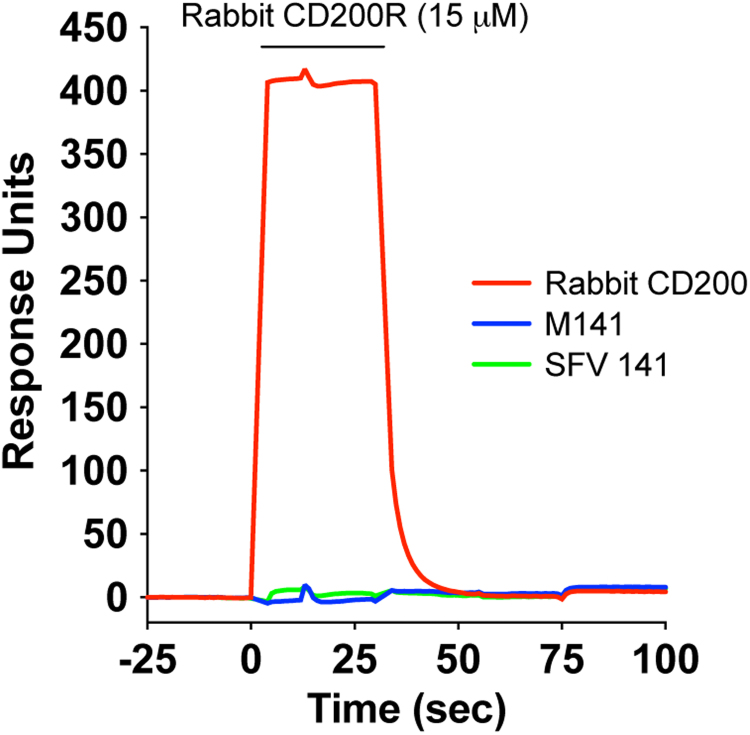
Rabbit CD200R does not bind poxvirus M141 proteins. Surface plasmon resonance analysis showing that rabbit CD200R (15 μM) bound to immobilised rabbit CD200 but not to myxoma M141 or rabbit fibroma virus (SFV141) proteins. The results are typical of four experiments.

No binding of rabbit CD200R to the recombinant myxoma M141 protein was observed by SPR (Fig. 3). The rabbit CD200R also failed to bind another rabbit poxvirus CD200-like sequence – rabbit fibroma virus SFV 141 even though high concentrations of recombinant proteins were used (15 µM). OX68 mAb that recognizes the CD4 portion of the chimeric proteins was also passed over the chip and bound to all four proteins giving at least 300 Response Units (RU) binding indicative of high levels of well folded chimeric protein being bound (data not shown).

**Fig. 4:**
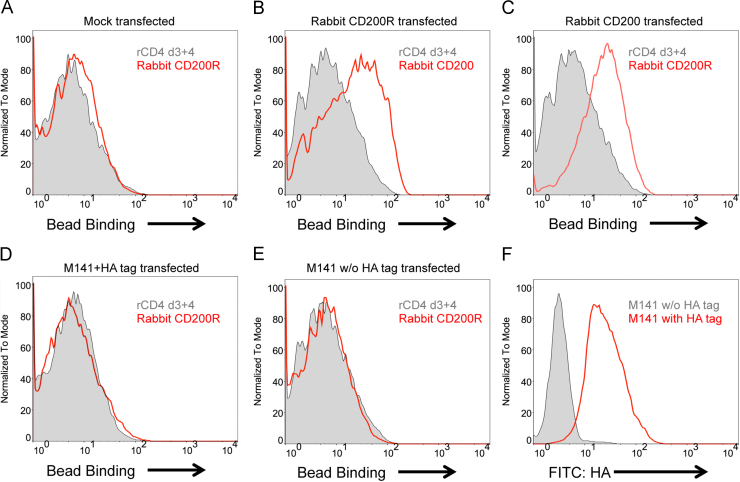
M141 protein is expressed at the cell surface but does not bind CD200R. (A–E) Stable 2B4 Reay cell lines transfected with mock vector (A) or vectors containing full length sequences of rabbit CD200R (B), rabbit CD200 (C), M141 with or without N terminal hemagglutinin tag (HA tag) (D–E) were tested for binding with green fluorescent beads coated with chimeric proteins containing the extracellular domains of rabbit CD200 or rabbit CD200R together with rCD4 d3+4 using flow cytometry. Beads coated with only rCD4 d3+4 were used as background control. (F) Surface expression of M141 protein on the HA tag transfected cell line was confirmed by staining the M141 with fluorescent HA tag antibody. A stable cell line expressing M141 without HA tag was used as background control for this experiment. Flow cytometry plots are representatives of three independent experiments.

To further exclude the possibility that the recombinant soluble viral proteins do not fold properly and thus cannot bind to the receptor, we generated cell lines that express full length viral proteins as well as rabbit CD200 and rabbit CD200R (Fig. 4). In the absence of specific reagents for these proteins, surface expressions of rabbit CD200 and rabbit CD200R were detected using fluorescent beads coated with rabbit CD200R and rabbit CD200 respectively by flow cytometry (Fig. 4B and C). Full length M141 was expressed with a hemagglutinin tag (HA tag) at the N terminus so that expression could be detected by staining the cells with anti HA antibodies (Fig. 4F). The tag is unlikely to affect ligand binding from analysis of the structure of the mouse CD200/CD200R complex (Hatherley et al., 2013) but an additional cell line expressing M141 without the HA tag was also generated. In order to detect low affinity interactions these cell lines were incubated with fluorescent beads coated with rabbit CD200R-rCD4d3+4 chimeric proteins (except for rabbit CD200R expressing cell line which was incubated with rabbit CD200 coated beads) or rCD4d3+4 coated beads as a control. Rabbit CD200R coated beads uniformly bound to the cells that expressed rabbit CD200 but failed to bind to any of the M141 expressing cells (Fig. 4A–E). This provided further evidence that the M141 protein, despite being expressed on the cell surface, does not interact with rabbit CD200R.

### Myxoma infected cells do not bind rabbit CD200R coated beads

**Fig. 5:**
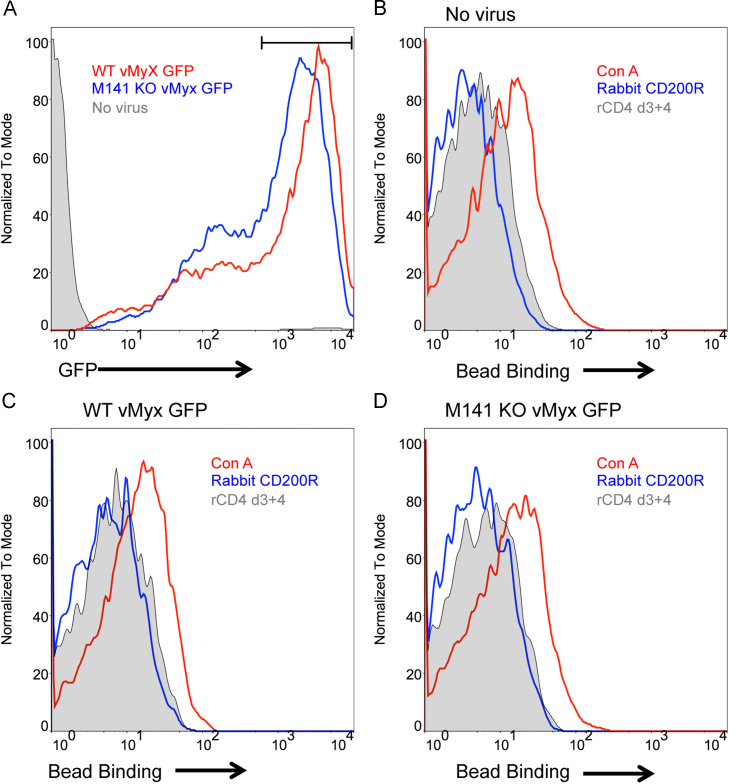
Flow cytometry showing rabbit CD200R coated beads do not bind myxoma infected cells. (A) RK13 cell lines infected with GFP tagged wild type (WT vMyx GFP) or GFP tagged M141 knock out (M141 KO vMyx GFP) virus were harvested 24 hpi. Both WT vMyx GFP (red) and M141 KO vMyx GFP (blue) infections generated comparable numbers of virus positive cells as shown by GFP expression in flow cytometry. Gray shaded area refers to uninfected cells. The gate indicates the GFP high population of the infected cells used for the following bead binding analyses. (B) Uninfected (C) WT vMyx GFP infected and (D) M141 KO vMyx GFP infected cells were tested for binding to nile red fluorescent beads coated with biotinylated rCD4 d3+4 chimeric proteins of rabbit CD200R (blue). Beads coated with biotinylated rCD4 d3+4 only (gray shaded) were used as negative control whereas, biotinylated concanavalin A coated beads were used as positive control for the bead binding assay. Flow cytometry plots are representatives of three independent experiments.

It is possible that the failure of M141 to interact with CD200R in both recombinant and cell surface expressing forms is due to additional viral proteins being required for binding. Therefore, we used a cell line (RK13) that is known to produce viable myxoma virus and to express M141 protein at the cell surface 12 h post-infection (Cameron et al., 2005). GFP labeled wild type (WT) or M141 knock out (KO) myxoma virus infected (0.5 moi) RK13 cells showed GFP fluorescence at similar levels (24 h) (Fig. 5A). This is in concordance with the previous observation that knocking out M141 does not alter the growth dynamics of the virus (Cameron et al., 2005). Uninfected RK13 cells and WT or M141 KO virus infected cells were tested for their ability to bind fluorescent beads coated with rabbit CD200R, rCD4d3+4 (negative control) and concanavalin A (Con A, positive control). Although all tested cells bound the positive control Con A beads, none bound the CD200R coated beads (Fig. 5B–D). This experiment was repeated using different virus levels (0.1 and 1 moi) and harvesting times (16 h and 30 h) but no binding with CD200R was observed (data not shown).

## Functional effects of M141 are not mediated by host CD200R

**Fig. 6:**
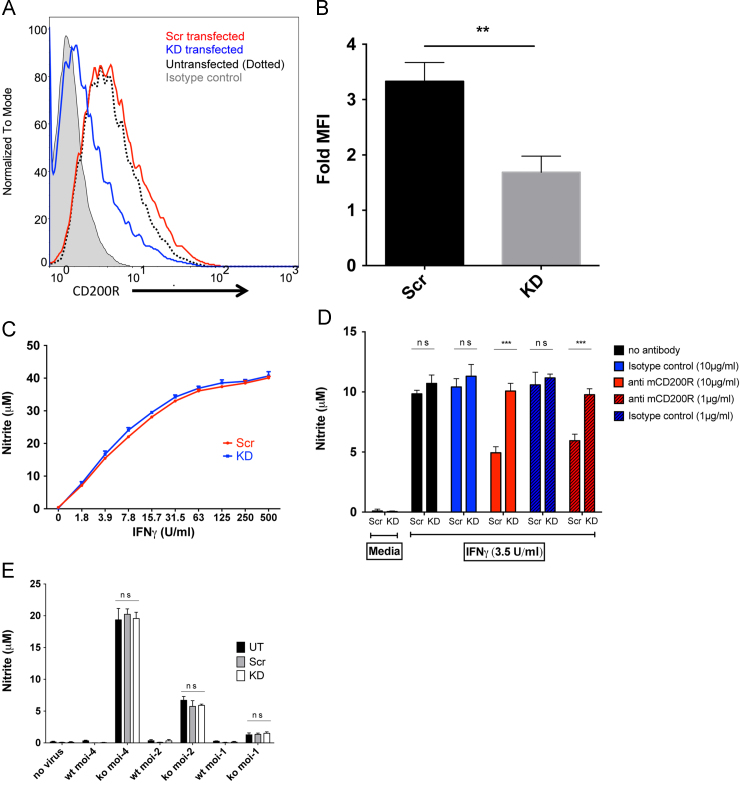
M141 related inhibition of nitrite production in RAW264.7 cells by myxoma infection is not mediated through CD200R. (A) Knock down of mCD200R expression by specific RNAi is shown by flow cytometry after labeling cells with mCD200R mAb (OX110 and OX131). Inhibition was obtained with the knock down vector (KD) but not the scrambled control (Scr). (B) Shows fold change in mean fluorescence intensity values (MFI) for Scr and KD stable cell lines from (A) (MFI of CD200R/ MFI of isotype control) (unpaired *T* test, *p*:0.0032; *n*=3) (C) Nitrite synthesis by RAW264.7 cells stimulated by soluble IFNγ is indistinguishable in the knock down and scrambled control. (D) Plate bound CD200R antibody gave inhibition of nitrite production in the control Scr cell line but not in KD cell line at two different concentrations of mAb (unpaired *T* test, *p*=0.0004 and 0.0008 respectively; *n*=3). (E) Differential production of nitrite by RAW264.7 cells following infection with either WT or KO myxoma virus is unaffected by knocking down CD200R. KD, Scr and UT cell lines were infected with wild type vMyx GFP (WT) or M141R KO vMyx GFP (KO) at 1, 2 and 4 multiplicity of infection (moi) and nitrite assayed. The wild type virus gave effective inhibition compared to the KO virus but the effect was the same in untransfected, scrambled or CD200R knockdown indicating that the inhibition is not dependent on the CD200R.

Although the M141 protein failed to bind to CD200R in various biochemical and cellular assays, it has been shown to have a potent inhibitory effect on various leukocyte functions (Cameron et al., 2005, Zhang et al., 2009). One of the most dramatic of these effects is the differential regulation of iNOS in virus infected RAW 264.7 cells. Cells infected with M141 KO virus upregulate iNOS expression while cells infected with WT virus fail to do so (Zhang et al., 2009). To test whether the differences in the RAW 264.7 cell activation upon virus infection are due to signaling through the inhibitory receptor CD200R, CD200R knockdown cells were generated by shRNA. The surface expression of CD200R was reduced compared to untransfected cells (UT) and cells transfected with control scrambled (Scr) shRNA sequence (Fig. 6A and B). The knockdown phenotype did not alter the cells׳ responsiveness to IFNγ (Fig. 6C), which was then used to test the functionality of the knockdown phenotype. CD200R can give inhibitory signals in RAW 264.7 cells, as crosslinking CD200R using plate bound CD200R mAb (OX131 (Akkaya et al., 2013)) gave clear inhibition of IFNγ induced NO production but not in the CD200R knockdown line (Fig. 6D). The possible role of CD200R in active infection by myxoma virus was tested as in Zhang et al. (2009) where NO production was induced by M141 KO virus but not wild type. This previous result was repeated and the effect was seen irrespective of whether the host RAW 264.7 cells were wild type, or transfected with shRNA against CD200R or a scrambled shRNA control (Fig. 6E). We conclude that the M141 protein acts on the innate immune system by a mechanism other than through CD200R.

## Discussion

The characterization of the CD200/CD200R interaction in rabbits indicates a similar system as found in many other species (Wright et al., 2003, Hatherley et al., 2005, Hatherley et al., 2013, Akkaya and Barclay, 2010). It also enabled the testing of the interactions of myxoma viral CD200-like protein with a host CD200R molecule. We were able to repeat the previous finding that myxoma virus lacking M141 could induce NO production in myeloid cells indicating a role in inhibiting myeloid cell activity. Although M141 shows sequence similarity to the ligand binding domain of CD200 (Fig. 1), we found no evidence that this inhibitory role of M141 involved the inhibitory receptor CD200R by various assays including direct binding, binding to transfected and infected cells, or by knockdown of the receptor. This distinguishes the poxvirus CD200-like protein from CD200 orthologues in herpesviruses where good evidence for direct binding and down regulation of cells have been obtained (Foster-Cuevas et al., 2004, Foster-Cuevas et al., 2011). Examination of the topology of the poxvirus CD200 like proteins (Fig. 1) and their sequences indicates that they differ more from the host than the herpesviruses. CD200 and CD200R both have two Ig-like domains and interact on opposing cells through their N-terminal domains. This requires the opposing membranes to be about 14 nm apart and consistent with many other interactions that occur at the immunological synapse (Hatherley et al., 2013, Barclay and Brown, 2006). In contrast the poxvirus CD200-like proteins only have one domain which would mean if they interacted with CD200R on opposing cells, the topology – i.e. distance between the cells – might not be optimal (see discussion of size in Hatherley et al. (2013)). In addition the Ig-like domain shows lower sequence identity with the host CD200 than the herpesvirus orthologues (summarized in Table 1). The key residues involved in the interface between mouse CD200 and mouse CD200R are indicated in Fig. 1B and it is notable that 9 out of 17 are conserved between all the mammalian sequences and the herpesviruses shown but only one of these residues is also conserved in the poxvirus sequences.

The evolutionary origins of the CD200-like sequences appear different between the herpesvirus and poxvirus CD200 like genes. In herpesviruses the sequences are more similar to the host species (around 40–87% see Table 1) indicating the genes have been acquired independently. The poxvirus sequences show lower identity with their host CD200 (around 20–30% identity) but in contrast the Ig-like domain of M141 shows higher sequence similarity to other poxviruses with around 50% amino acid sequence identity to deerpox, sheeppox, lumpy skin disease virus and 70% identity to rabbit fibroma virus proteins. This suggests that the poxvirus sequence may have been acquired, evolved and maintained by poxviruses. As the poxvirus CD200-like proteins seem unlikely to bind CD200R, it remains a puzzle as to how they function. We suggest M141 is no longer termed CD200-like. The finding that the rabbit myxoma product has profound cross species functional effects on mouse RAW 264.7 cells (Zhang et al., 2009) suggests it is interacting with a relatively well conserved receptor.

## Materials and methods

### Production of recombinant proteins

The complete coding sequences of rabbit CD200 and CD200R were identified from Ensembl database and the sequences were submitted to the NCBI database under accession numbers BK009358, BK009359 respectively. The sequence corresponding to the extracellular regions (residues 1-238 for CD200R and 1-233 for CD200) were codon-optimized, synthesized (Geneart Gene Synthesis, Invitrogen) and cloned into pEE14 together with a hexahistidine tag for expression in CHO-K1 cells (Hatherley et al., 2005). These regions were also cloned into pEF-BOS for transient expression in 293T cells as chimeric proteins with rat CD4 domains 3 and 4 (rCD4d3+4) and a biotinylation sequence (Akkaya and Barclay, 2013, Brown et al., 1998). Proteins used for kinetic analysis were further purified by gel filtration to ensure monomeric protein using a Superdex 75 16/60 column (GE Life Sciences) and AKTA FPLC at 4 °C. Full length coding sequences of myxoma M141 (Accession no: NP_051855.1) and rabbit fibroma virus (SFV) 141 (Accession no: NP_052027.1) were similarly codon optimized and synthesized by Geneart Gene Synthesis. The extracellular regions of the proteins (residues 1-178 for M141 and 1-199 for SFV 141) were cloned into pEF-BOS vector for generation of soluble chimeras with rCD4d3+4 and a biotinylation signal as described above.

### Measurement of the affinity of the rabbit CD200/CD200R interaction using surface plasmon resonance

Surface plasmon resonance experiments were performed using a BIAcore 3000. All experiments were performed at 37 °C using 10 mM HEPES pH 7.5, 150 mM NaCl, 3 mM EDTA, 0.02% P20 (HEPES-EP) as running buffer. Concentrated tissue culture supernatants containing biotinylated rCD4d3+4 chimeras with the extra cellular domains of rabbit CD200, rabbit CD200R, and rat CD200R were immobilised to streptavidin coated flow cells of a CM5 chip (together with rCD4 d3+4 as a negative control). Purified monomeric fractions of soluble recombinant rabbit CD200 or CD200R proteins were passed over the flow cells in increasing concentrations. Equilibrium binding was calculated by subtracting the control binding values from the ligand-receptor binding values and the affinity calculated using Langmuir binding isotherm formula and GraphPad PRISM (GraphPad Software Inc.).

### Cell lines and viruses

RK13 rabbit kidney cell line, 293T human embryonic kidney cell line, CHO-K1 chinese hamster ovary cell line and RAW 264.7 mouse macrophage cell lines were obtained from European Collection of Cell Cultures (Salisbury, UK). 2B4 Reay T cell hybridoma, Phoenix Eco packaging cell line were from Marion H. Brown and as described in Akkaya et al. (2013). All cell lines were grown in RPMI 1640 (Gibco) media supplemented with 50 µM 2-mercaptoethanol, 50 U/ml penicillin, 50 µM streptomycin (PAA), 2 mM L-glutamine (PAA), 0.1 mM non-essential amino acids (Sigma), 1 mM sodium pyruvate (Sigma), 10 mM HEPES (PAA). WT vMyx GFP and M141KO vMyx GFP viruses were gifts from Grant McFadden (University of Florida) and were as described previously in Cameron et al. (2005) and Zhang et al. (2009). Viral stocks for both viruses were grown and purified using the guidelines in Smallwood et al. (2010).

## Bead binding to cell lines

Full length rabbit CD200, rabbit CD200R, M141R genes (with and without 5′ HA tag) were cloned into pFB-Neo retroviral expression vector (Agilent), transfected into Phoenix Eco packaging cell line using Fugene 6 (Promega) and the supernatants containing retroviruses harvested and used to transduce the 2B4 Reay T cell line. Stable cell lines were selected with G418 and tested for binding with green fluorescent beads (Spherotech) coated with rCDd3+4 chimeric proteins of rabbit CD200 and CD200R, or rCD4d3+4 (control). Expression of HA tag conjugated M141 molecule was tested using FITC conjugated anti-HA mAb (Sigma).

RK13 cells were infected with either WT (WT vMyx GFP) or M141 KO (M141 KO vMyx GFP) virus (0.5 moi). 24 h post infection cells were harvested and tested for their binding with nile red fluorescent beads (Spherotech) coated with rCDd3+4 chimeric proteins of rabbit CD200R or mouse CD200R. Beads coated with rCD4d3+4 were used as a background control whereas beads coated with concanavalin A (Vector Laboratories) were used as a positive control. Dead cells were gated out by staining the cells with LIVE/DEAD Dead cell staining kit (Invitrogen). Flow cytometry experiments were performed using a CyAn™ flow cytometer (Beckman Coulter) and data analysis was performed using Flow Jo software.

## mCD200R knock down

Specific shRNA against host CD200R and non specific scrambled shRNA were generated using the following oligonucleotides designed by the oligoengine RNAi design tool: (Scrambled forward: 5′GATCCCCGAACCATATCTATACTGAATTCAAGAGATTCAGTATAGATATGGTTCTTTTTC3′; scrambled reverse: 5′TCGAGAAAAAGAACCATATCTATACTGAATCTCTTGAATTCAGTATAGATATGGTTCGGG3′; specific forward: 5′GATCCCCCCAAAATTAGAAGCTACTTTTCAAGAGAAAGTAGCTTCTAATTTTGGTTTTTC3′; specific reverse: 5′TCGAGAAAAACCAAAATTAGAAGCTACTTTCTCTTGAAAAGTAGCTTCTAATTTTGGGGG3′). Forward and reverse oligonucleotides for each group were mixed at equimolar concentrations and annealed by stepwise cooling. The annealed inserts were ligated into the pSUPERIOR Puro (Oligoengine) vector according to manufacturer׳s guidelines. Vectors containing specific or scrambled inserts were transfected into RAW 264.7 cell line using Fugene 6 transfection reagent (Promega) and stable colonies were generated by growing the cells in 4 μg/ml puromycin containing media. Surface levels of CD200R on transfected cells were determined by staining the cells with a mCD200R mAb (OX110) or an isotype control.

### RAW 264.7 cell stimulation and Griess assay

To test the functional relevance of CD200R knock down, transfected (scrambled and specific) RAW 264.7 cells were seeded in 96 well plates (1.2×10^5^ live cells in 200 μl media with or without added IFN gamma) previously coated with mCD200R monoclonal antibody (OX131) or an isotype control antibody for 16 h. To test the effect of the possible M141/CD200R interactions RAW 264.7 cells (transfected and untransfected) plated similarly on uncoated 96 well plates were stimulated by infecting with myxoma virus (WT vMyx GFP) or M141 KO (M141 KO vMyx GFP) virus) for 16 h as previously described in Zhang et al. (2009). Supernatants were collected and assayed for nitrite using the Griess Reagent System (Promega).

## Figures and Tables

**Table 1:** Amino acid sequence identity between domain one of CD200 from the species indicated and herpes and poxvirus CD200-like sequences. Data and abbreviations are from analysis in Fig. 1.

	SFV gp141	MV M141	LSDV 138	YLDV 141	HHV8 K14	RCMV e127	mCD200	ratCD200	rabCD200	hCD200
SFV gp141	100									
MV M141	72	100								
LSDV 138	39	44	100							
YLDV 141	40	42	53	100						
HHV8 K14	25	26	28	25	100					
RCMV e127	20	22	30	29	44	100				
mCD200	22	26	29	29	40	80	100			
ratCD200	20	22	28	27	41	87	90	100		
rabCD200	21	24	29	28	40	72	78	76	100	
hCD200	24	25	28	28	41	71	79	77	84	100
